# Anatase Incorporation to Bioactive Scaffolds Based on Salmon Gelatin and Its Effects on Muscle Cell Growth

**DOI:** 10.3390/polym12091943

**Published:** 2020-08-28

**Authors:** Cristian A. Acevedo, Yusser Olguín, Nicole Orellana, Elizabeth Sánchez, Marzena Pepczynska, Javier Enrione

**Affiliations:** 1 Centro de Biotecnología, Universidad Técnica Federico Santa María, Avenida España 1680, Valparaíso 2340000, Chile; cristian.acevedo@usm.cl (C.A.A.); nicole.orellana@usm.cl (N.O.); elizabeth.sanchez@usm.cl (E.S.); 2 Centro Científico Tecnológico de Valparaíso CCTVaL, Universidad Técnica Federico Santa María, Avenida España 1680, Valparaíso 2340000, Chile; yusser.olguin@usm.cl; 3 Departamento de Física, Universidad Técnica Federico Santa María, Avenida España 1680, Valparaíso 2340000, Chile; 4 Escuela de Nutrición y Dietética, Facultad de Medicina, Universidad de los Andes, Monseñor Álvaro del Portillo 12455, Las Condes, Santiago 7550000, Chile; mpepczynska@uandes.cl; 5 Biopolymer Research and Engineering Lab., Center for Biomedical Research and Innovation (CIIB), Universidad de los Andes, Monseñor Álvaro del Portillo 12455, Las Condes, Santiago 7550000, Chile

**Keywords:** anatase, muscle cells, scaffold, salmon gelatin, tissue engineering

## Abstract

The development of new polymer scaffolds is essential for tissue engineering and for culturing cells. The use of non-mammalian bioactive components to formulate these materials is an emerging field. In our previous work, a scaffold based on salmon gelatin was developed and tested in animal models to regenerate tissues effectively and safely. Here, the incorporation of anatase nanoparticles into this scaffold was formulated, studying the new composite structure by scanning electron microscopy, differential scanning calorimetry and dynamic mechanical analysis. The incorporation of anatase nanoparticles modified the scaffold microstructure by increasing the pore size from 208 to 239 µm and significantly changing the pore shape. The glass transition temperature changed from 46.9 to 55.8 °C, and an increase in the elastic modulus from 79.5 to 537.8 kPa was observed. The biocompatibility of the scaffolds was tested using C2C12 myoblasts, modulating their attachment and growth. The anatase nanoparticles modified the stiffness of the material, making it possible to increase the growth of myoblasts cultured onto scaffolds, which envisions their use in muscle tissue engineering.

## 1. Introduction

Tissue engineering uses therapeutic alternatives that allow the functional regeneration of damaged tissues, mainly through the inclusion of materials and cells in the affected areas, providing factors for cell proliferation and tissue repair [1]. The materials used to culture cells for tissue engineering require specific characteristics and components of the extracellular matrix (ECM) to treat the injured tissue [2]. The microstructure of these biomaterials, particularly the way in which some of their features (e.g., pores) are distributed and interconnected, together with the type of synthesis (e.g., chemical crosslinking) [3] used in the fabrication, contribute to the establishment of the scaffold concept in tissue engineering [4], which not only supports the deposition of cells, but also maintains active properties that enable cell adhesion, proliferation, and differentiation [5].

Gelatin is a biomaterial with outstanding and well-known physical properties, which include high biodegradability and biocompatibility, and combined with collagen, they have generated promising results through a variety of tissue engineering formulations [6]. The source of the extracted gelatin establishes characteristics that affect the scaffold properties [7,8].

Fish are a promising source of gelatin for the development of novel scaffolds [9,10]. In particular, gelatin from salmon skin, like other cold-water fish gelatins, has a lower concentration of imino acids (proline and hydroxyproline) and a lower molecular weight distribution, showing significant differences in thermal and viscoelastic properties compared with mammalian (bovine or porcine) and warm-water fish gelatins, providing some advantages for scaffold fabrication [11,12].

When considering new biomaterials formulated based on salmon gelatin, one of the least studied areas corresponds to the development of scaffolds for muscle cells, where the use of mammals gelatins is common [13]. For the development of scaffolds for muscle tissue engineering (MTE), the materials need complementary properties that can support the development of muscle cells [14], where the structure and mechanical properties, such as the stiffness of the material, are key variables to be considered [15,16,17,18].

Titanium oxide (TiO_2_) nanostructures have been used to culture muscle cells (C2C12 myoblasts) [19] and other cells, such as fibroblasts and keratinocytes, showing adequate adhesion and cell proliferation for tissue engineering [20]. TiO_2_ is spontaneously formed from titanium in the air and electrolytes; it is stable in the body and does not degrade. TiO_2_ with specific crystal structures, such as anatase, is effective in in vitro apatite formation, which is believed to be a prerequisite for bioactivity [21]. In particular, the anatase form of TiO_2_ is widely accepted as a component that adds functionality to scaffolds due to its demonstrated biocompatibility with various cell types [22,23], including muscle cells [24]. TiO_2_ nanostructures can be used to affect the cell response and to influence cell fate in tissue engineering. Over the last two decades, several in vitro studies have focused on the interaction of TiO_2_ nanostructures with different kinds of cells, such as chondrocytes, endothelial cells, smooth muscle cells, macrophages, mesenchymal stem cells, neural progenitors, osteoblasts, periodontal ligament stem cells, platelets, and leucocytes [25]. The design of nano-anatase polymeric scaffolds offers an exciting approach to combine the advantages of a degradable polymer with those of nanoparticles to optimize physical and biological properties for regeneration [21].

In our previous work [11,12], a novel porous scaffold based on salmon gelatin and excipients (chitosan, agarose, and glycerol) was developed and tested in animal models (rabbits and pigs), with excellent results, to regenerate tissues. It is of great interest to us to continue studying this biomaterial, and due to the high use of anatase for tissue engineering, we researched the incorporation of anatase nanoparticles into this scaffold and its effect on myoblast cell adhesion and growth.

## 2. Materials and Methods 

### 2.1. Scaffold Preparation

Gelatin was extracted from salmon skins according to the methodology described by Enrione et al. [12]. Chitosan (pharmaceutical grade, 95% deacetylated, 300 kDa, derived from crab shells) was purchased from Quitoquimica (Concepción, Chile). Agarose (molecular biology grade) was purchased from Lonza (Morristown, NJ, USA). Glycerol (pharmaceutical grade) was purchased from Merck (Darmstadt, Germany). EDC (*N*-ethyl-*N*’-(3-dimethylaminopropyl)carbodiimide hydrochloride), NHS (*N*-hydroxysuccinimide), MES (2-(*N*-morpholino)ethanesulfonic acid hydrate), and anatase nanoparticles (nanoparticle size < 25 nm) were purchased from Sigma-Aldrich (St. Louis, MO, USA).

The salmon-gelatin-based scaffolds (with excipients: chitosan, agarose, and glycerol) were fabricated using our previously reported method [11,12], with minimal modifications to incorporate anatase (nanoparticles) into the composite. The anatase nanoparticles were previously dissolved into a chitosan stock solution (2% w/v in 1% w/v acetic acid). Salmon gelatin, agarose, and glycerol stock solutions were prepared in Milli-Q water (2% w/v, 0.4% w/v, and 1% w/v, respectively). Briefly, the stock solutions were used to prepare three composite solutions with different anatase concentrations: salmon gelatin, 0.6% w/v; chitosan, 0.2% w/v; agarose, 0.2% w/v; glycerol, 0.1% w/v; and anatase, 0.0, 0.1, and 0.2% w/v. The solutions were mixed at 50 °C for 1 h and poured into Petri dishes (adjusting the volume to obtain a height of 3 mm). They were then were cooled at 4 °C, frozen at −80 °C, and lyophilized. The dry composites were crosslinked using EDC/NHS/MES/ethanol (30 mM/8 mM/50 mM/90% v/v) at room temperature for 3 h. The resultant crosslinked composites were washed (pure ethanol; ethanol, 70% v/v; ethanol, 40% v/v; and water), frozen at −80 °C, and freeze-dried to obtain the scaffolds. The scaffolds were stored with silica gel until experimentation.

### 2.2. Scaffold Microstructural Characterization

The scaffold microstructure was analyzed by SEM/EDS (scanning electron microscopy/energy dispersive X-ray spectroscopy). Prior to the measurements, the samples were coated with gold (10–20 nm). The coated scaffolds were scanned by a Carl Zeiss SEM (EVO MA 10, Oberkochen, Germany) system equipped with EDS (X-Act, Oxford Instruments, Abingdon, UK).

The pore size (equivalent circular diameter) and pore shape (circularity values equal to 1 and 0 for a perfect circle and irregular elongated shape, respectively) of the scaffolds were determined from the SEM images using ImageJ software (NIH, Bethesda, MD, USA). Three SEM images per scaffold were used, counting at least 100 pores for each formulation. The equations used for pore size and shape were [3,10,12]:

(1) $$Pore\;size\left(equivalent\;circular\;diameter\right)=\frac{\sqrt{4A}}{\sqrt{\pi }}$$

(2) $$Pore\;shape\;\left(circularity\right)=\frac{4A\pi }{P^{2}}$$

where *A* and *P* are the area and perimeter of the pore, respectively.

### 2.3. Differential Scanning Calorimetry (DSC)

A differential scanning calorimeter (DSC 1 STAR System, Mettler Toledo, Greifensee, Switzerland) with an intracooler TC100 (Huber, Offenburg, Germany) was used to characterize the scaffolds. The measurements were carried out using ~10 mg of sample in a stainless-steel pan (120 µL). An empty pan was used as a reference. The thermal scanning protocol used was: cooling down from 25 to 0 °C at 40 °C/min, isothermic step at 0 °C for 5 min, and heating to 150 °C at 10 °C/min. The samples were subjected to the same thermal protocol twice. The melting temperature (*T*_m_) and changes in the enthalpy of melting (∆*H*_m_) were determined from the first scan. The glass transition temperature (*T*_g_) was determined in the amorphous material (second scan). The curves were analyzed using the STARe software (DB V 12.10). Prior to the measurements, the melting temperature and enthalpy values were calibrated using indium as standard. All determinations were made in triplicate.

### 2.4. Dynamic Mechanical Analysis (DMA)

The mechanical properties of the scaffolds were measured using a dynamic mechanical analyzer (DMA 1 Star System, Mettler Toledo, Greifensee, Switzerland) equipped with two parallel compression plates. Before conducting the experiments, all instrumental calibrations were performed. The compression frequencies applied were 1, 5, and 10 Hz and were tested from 0 to 120 °C at a heating rate of 3 °C/min. The displacement amplitude used was 10 µm. Liquid nitrogen was used as a cooling medium. The geometries of the sample were cylinders with a height and diameter of ~2 mm and ~10 mm, respectively. All determinations were made in triplicate.

### 2.5. Cell Culture

The cell line C2C12 was used as a model of myoblast cells. The cell line was purchased from the European Collection of Authenticated Cell Cultures (ECACC) and supplied by Sigma-Aldrich (St. Louis, MO, USA). The cells were cultured using standard conditions for cell culture (37 °C and 5% CO_2_). The cells were seeded onto the scaffold at 1 × 10^4^ cells/cm^2^. Scaffold sections of 1 cm^2^ were used to seed the cells. The medium used to culture the cells was DMEM high glucose (Gibco, Life Technologies, Grand Island, NY, USA), supplemented with 10% fetal bovine serum, L-glutamine (2 mM), and antibiotics (100 U/mL of penicillin and 100 µg/mL of streptomycin). Cell adhesion and growth onto the scaffolds were performed in triplicate as described below.

The cell adhesion and cell growth were quantified by estimating the viable biomass in the scaffold at different times by using the commercial colorimetric assay WST-1 (Roche, Mannheim, Germany). Cell adhesion was assessed after 4 h of seeding and compared with the control (cells adhered to commercial plastic for cell culture). For cell growth, the membranes were sampled at 24, 48, and 72 h; then, the data were fitted using the classic exponential model to obtain the specific growth rate (µ) [26].

### 2.6. Statistical Analysis

The data were analyzed using one-way analysis of variance (ANOVA) and considered to be significantly different when *p* < 0.05. The analysis was performed using Excel (version 16.4, Microsoft).

## 3. Results and Discussion

### 3.1. Microstructure of the Scaffolds

**Figure 1:**
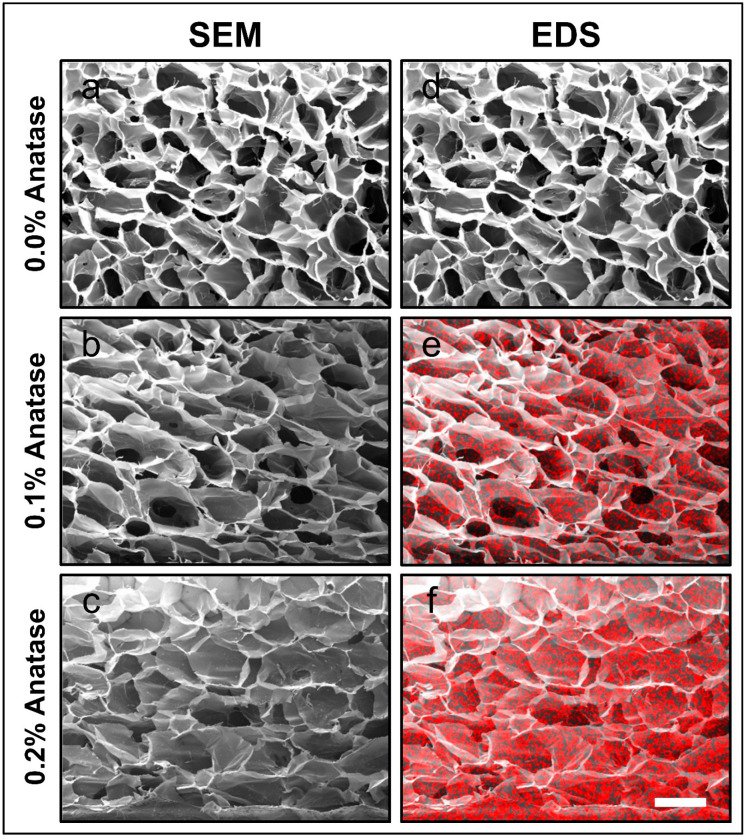
Microstructure of the scaffolds prepared with different anatase concentrations. The images were obtained with SEM (**a**–**c**). The red spots show the titanium (Ti) mapping with EDS (**d**–**f**), indicating a homogeneous distribution of anatase (TiO_2_) nanoparticles. Bar scale 300 μm.

The microstructure of the scaffolds is shown in Figure 1a–c. The incorporation of anatase altered the pore morphology (size and shape) when the concentration of anatase increased to 0.2%. The changes in pore size and shape were significant (*p* < 0.05, ANOVA) in both cases. Indeed, changes in pore size from 208.4 to 239.5 µm and shape (circularity) from 0.805 to 0.691 are reported in Table 1. This effect could be related to an increase in viscosity of the polymer solutions due to the inclusion of anatase nanoparticles [27]. This increase in viscosity would affect the pore structure formation and size relaxation upon cooling, which in the case of the freeze-drying of the samples would increase the pore size after drying [28]. The pore changed from a round-like geometry to a flatter shape, which could be explained by a dentification effect of the added anatase since its higher relative density can increase the overall material weight, showing an ellipsoidal-like geometry parallel to the surface [29]. This modulation of the pore size and shape is fundamental in order to provide a favorable environment for cell survival and growth [30], which in the case of muscle cells is essential for interconnectivity and three-dimensional proliferation [31].

As indicated in Section 2.2, the SEM analysis was complemented by an EDS analysis, allowing for the mapping of titanium (Ti) as red dots over the scaffold structure. Figure 1d–f depicts a homogeneous distribution of anatase (TiO_2_) nanoparticles on the material. It is important to highlight that the homogeneity of the particle dispersion in the scaffold was obtained without the use of surfactants, which tend to generate negative biological interactions and modify the structural characteristics of the gelatin in the formulation of the scaffolds [32,33,34,35].

The results shown in Table 1 also indicate that the modification of the microstructure of the scaffolds was dependent on the anatase concentration in the composites.

As already discussed, the incorporation of nanoparticles into scaffolds is done with the aim of improving their mechanical and pore surface capabilities [36], but they can often modify the thermophysical properties of the polymers present in the composites during the manufacturing processes of the biomaterial [11].

### 3.2. Thermal Properties of the Scaffolds

The analysis of the thermal properties of the scaffolds by differential scanning calorimetry is shown in Table 1. It can be observed that the incorporation of anatase nanoparticles modified the thermal parameters when their concentration was increased. In particular, the glass transition temperature (*T*_g_) increased significantly (*p* < 0.05, ANOVA) from 46.9 to 55.8 °C and the melting temperature (*T*_m_) increased from 68.5 to 73.2 °C (*p* < 0.05, ANOVA). This increase in both parameters can be explained by the change in molecular mobility of the polymer mixes, which would be reduced by the addition of anatase. The scaffold with anatase nanoparticles would require higher levels of energy for both transitions to occur. The Δ*H*_m_ decrease (*p* < 0.05, ANOVA) when anatase was added to the scaffolds was associated with the disruption of a lower number of triple helices of the gelatin fraction formed upon cooling during the scaffold fabrication [37]. Lower values of Δ*H*_m_ would represent a lower number of triple helices formed due to the increase in the viscosity of the system [38].

### 3.3. Mechanical Properties of the Scaffolds

**Figure 2:**
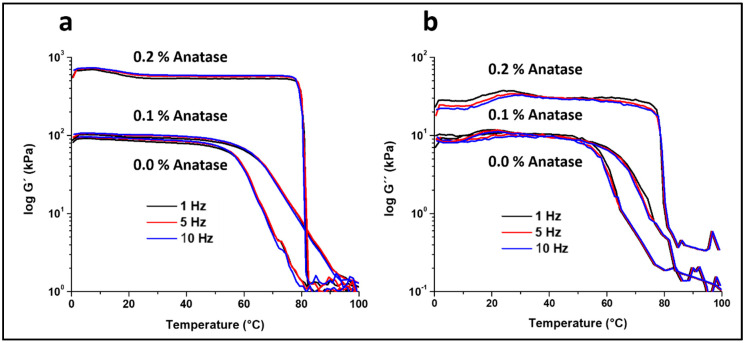
Results of the dynamic mechanical analysis (DMA) at 1, 5, and 10 Hz. (**a**) Elastic modulus (G’). (**b**) Loss modulus (G’’).

The elastic modulus (G’) values of the formulated scaffolds at the three frequencies from DMA are depicted in Figure 2a. The G’ values of the control scaffold were relatively constant at 85–95 kPa at temperatures below 50 °C, after which the G’ decreased steadily to the lowest value at 80 °C. This decrease in G’ was related to the melting of the amorphous fraction of the gelatin in the composite. It is also clear in this figure that anatase affects the mechanical properties of the scaffolds [39]. The scaffold with 0.1% anatase showed similar G’ values as the control below 60 °C; however, at higher temperatures, G’ decreased to the lowest value at a temperature of ~100 °C. It was interesting to note that the slope of the drop in G’ with temperature was less steep for the 0.1% anatase sample, and therefore, at temperatures higher than 60 °C, the values of G’ were significantly higher for the 0.1% anatase scaffold. For instance, at 70 °C, G’ was 8 kPa for the control scaffold and 40 kPa for the scaffold containing anatase.

In the case of the scaffold with 0.2% anatase, the values of G’ were several times higher, reaching ~600 kPa for the temperature range from 0 to 80 °C. Table 2 shows the values of G’ at 37 °C as a reference temperature for the three formulations. The increase of G’ at 37 °C was significant (*p* < 0.05, ANOVA) in all the frequencies tested. At temperatures higher than 80 °C, the modulus dropped almost vertically to similar values of the other samples. This drop in G’ can be explained by the reinforcing effect of the anatase nanoparticles of the amorphous fraction of the composite, sustaining the compression up to a temperature near melting of the composite (Table 1), at which the material collapsed. The improvement in mechanical properties by anatase can be explained by the filling effect within the gelatin-chitosan-agarose composite by the anatase nanoparticles [39], significantly increasing the elastic components (stiffness) of these materials.

Similar changes in mechanical properties were observed for all the frequencies tested, reporting a slight increase in G’ values from 1 to 10 Hz (Table 2), which was expected since the mechanical response in this type of material should be time-dependent.

Figure 2b shows the loss modulus (G’’) with temperature from 0 to 100 °C. The curve profiles for all the samples were similar to those observed for G’. The values of G’’ remained relatively constant up to a marked drop in its values, which occurred at ~55, 65 and 80 °C for the control, 0.1% anatase, and 0.2% anatase samples, respectively. It is important to note that the G’’ values for all the samples were lower than the G’ values, and that the values of tan δ were relatively constant and lower than 0.2 (data not shown). These results indicate that the structure of the samples was stable throughout the temperature range and frequencies tested, and the drop in G’ and G’’ was related to the melting of crystalline fractions in the composite rather than the relaxation of amorphous components.

These results show that the mechanical properties, the elastic modulus and the loss modulus, of the formulations were very stable throughout a wide range of temperatures. This behavior was particularly clear for the scaffold with 0.2% anatase, with an almost constant value of G’ at a temperature of 80 °C. However, it is important to state that in future work, similar tests should be performed under hydrated conditions for more realistic information on the mechanical behavior of the scaffolds in physiological-like conditions.

### 3.4. Behavior of Myoblasts Cultured into Scaffolds

The biological behavior was evaluated by measuring the adhesion and growth of C2C12 myoblasts seeded onto the scaffolds (Table 3). The cell adhesion measured after four hours of seeding showed a concentration-dependent relationship with anatase. The adhesion changed significantly (*p* < 0.05, ANOVA) when anatase was added. It is known that the cell adhesion onto scaffolds depends on the activity of the binding receptors αvβ3 and α5β1 that can recognize gelatin RGD sequences [40,41]. The inclusion of nanoparticles likely reduces adhesion by covering RGD sequences—a known situation that has been studied in RGD peptides coating [42,43].

The inclusion of anatase into scaffolds induced a change in the pore morphology, increasing the size and producing a deformation of the pore (circularity decreased with anatase, see Table 1), which could be related to the adhesion of myoblasts [44,45]. It has been reported that the adhesion of myoblasts and fibroblasts onto gelatin-chitosan scaffolds is close to 40% [46,47,48], its value being comparable to our lowest data obtained (0.2% anatase). Besides, the pore size of gelatin-based scaffolds affects the adhesion and growth of the cells [46], and scaffold microstructural changes can decrease adhesion and increase proliferation simultaneously [47], which is considered an improvement for tissue engineering purposes.

**Figure 3:**
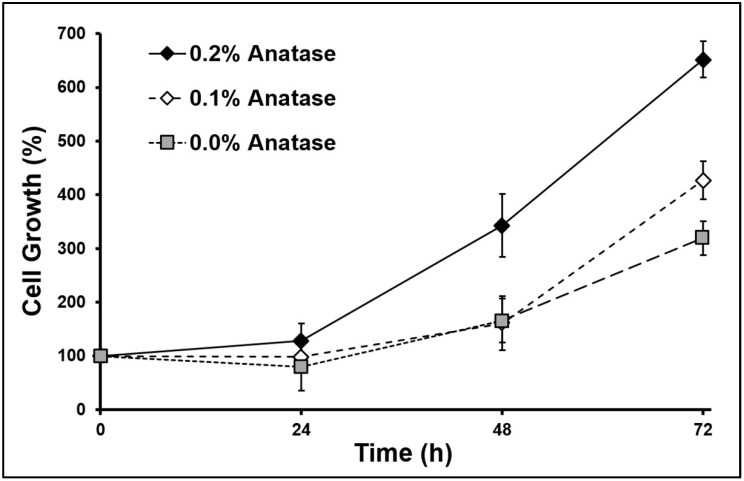
Cell growth of C2C12 myoblasts cultured onto scaffolds at 1 × 10^4^ cell/cm^2^.

The kinetics of cell growth are shown in Figure 3, indicating a lag phase on the first day, and then an exponential growth in all scaffolds. The specific cell growth (μ) showed a significant increase (*p* < 0.05, ANOVA) when the anatase concentration increased (see Table 3). Particularly in muscle cells, the proliferative activity depends on the form of TiO_2_ [20]. It is considered a bioactive coating that can enhance proliferation and differentiation [49,50], mainly in the anatase form of TiO_2_ [51]. On the other hand, the increase of cell growth correlates positively with the increase of scaffold stiffness (see G’ in Table 2). It has been reported that scaffold stiffness is an important physical factor in the response of many cell lineages, including myoblasts and other mesenchymal cells [16]. The behavior of muscle cells has a strong dependence on the stiffness, affecting adhesion, spreading, growth, and differentiation [15,16,17]. Porosity and stiffness are two important factors involved in the cell behavior [18], which changed together by incorporating anatase into our scaffolds, modulating the adhesion and growth of the myoblasts cultured onto them.

The non-mammalian scaffold developed here, made mainly with salmon gelatin, shows a high biocompatibility and capacity to incorporate anatase nanoparticles, which can be used to modulate the response of cell adhesion and growth in the field of tissue engineering.

## 4. Conclusions

The development of scaffolds for tissue engineering requires the exploration of different polymeric alternatives and their combination with biological, chemical, and physical elements to provide specific functions for each tissue. In this work, we show for first time the physical and biological characterization of a scaffold based on salmon gelatin and anatase nanoparticles, which showed biocompatibility with myoblasts. The thermal and mechanical behavior of the salmon-gelatin-based scaffold was modified by the incorporation of anatase nanoparticles, increasing the myoblast growth on the scaffold. The increase of stiffness and cell growth produced by the incorporation of anatase nanoparticles could be used to improve scaffold design for muscle tissue engineering.

## Figures and Tables

**Table 1:** Microstructure by SEM and thermal characterization by DSC of the scaffolds prepared with different anatase concentrations.

Anatase (%)	Pore Size (µm)	Pore Shape (Circularity)	*T*_g_ (°C)	*T*_m_ (°C)	Δ*H*_m_ (J g^−1^)
0.0	208.4 ± 22.0	0.805 ± 0.069	46.9 ± 1.0	68.5 ± 3.0	8.1 ± 0.9
0.1	223.6 ± 28.0	0.743 ± 0.070	55.9 ± 0.5	72.3 ± 0.5	5.5 ± 0.1
0.2	239.5 ± 21.2	0.691 ± 0.112	55.8 ± 1.6	73.2 ± 1.1	5.3 ± 0.5

**Table 2:** Elastic modulus (G’) and loss modulus (G’’) at 37 °C of the scaffolds prepared with different anatase concentrations.

Anatase (%)	Elastic Modulus (G’) at 37 °C (kPa)			Loss Modulus (G’’) at 37 °C (kPa)		
	1 Hz	5 Hz	10 Hz	1 Hz	5 Hz	10 Hz
0.0	79.5 ± 4.7	84.0 ± 3.9	85.5 ± 4.0	10.3 ± 0.33	10.4 ± 0.35	9.6 ± 0.41
0.1	90.5 ± 6.2	95.5 ± 6.8	97.4 ± 4.8	9.8 ± 0.58	9.7 ± 0.48	9.7 ± 0.21
0.2	537.8 ± 3.9	572.3 ± 4.4	± 4.8	31.6 ± 0.42	31.2 ± 0.51	30.0 ± 0.32

**Table 3:** Cell adhesion and growth (µ) on scaffolds prepared with different anatase concentrations.

Anatase (%)	Adhesion (%)	µ (d^−1^)
0	62.4 ± 4.69	0.692 ± 0.021
0.1	57.42 ± 3.86	0.734 ± 0.077
0.2	37.26 ± 2.75	0.813 ± 0.078
Control (commercial plastic)	100.00 ± 2.46	0.926 ± 0.042
